# Redirection and reshaping of intense extreme-ultraviolet radiation

**DOI:** 10.1126/sciadv.aef5300

**Published:** 2026-05-29

**Authors:** Yu He, Alexander Magunia, Harijyoti Mandal, Muwaffaq Ali Mourtada, Carlo Kleine, Arikta Saha, Marc Rebholz, Gergana D. Borisova, Lina Hedewig, Hannes Lindenblatt, Florian Trost, Ulrike Frühling, Christina C. Papadopoulou, Elisa Appi, Stefan Düsterer, Tino Lang, Skirmantas Alisauskas, Christoph M. Heyl, Steffen Palutke, Markus Braune, Christina Bömer, Dietrich Krebs, Doriana Vinci, Philip Mosel, Peer Biesterfeld, Ingmar Hartl, Robert Moshammer, Milutin Kovacev, Kiyoshi Ueda, Mette B. Gaarde, Christian Ott, Thomas Pfeifer

**Affiliations:** ^1^ Max-Planck-Institut für Kernphysik, Saupfercheckweg 1, 69117 Heidelberg, Germany.; ^2^ Deutsches Elektronen-Synchrotron DESY, Notkestraße 85, 22607 Hamburg, Germany.; ^3^ Department of Physics, Lund University, SE-221 00 Lund, Sweden.; ^4^ GSI Helmholtzzentrum für Schwerionenforschung GmbH, Planckstraße 1, 64291 Darmstadt, Germany.; ^5^ Helmholtz Institute Jena, Fröbelstieg 3, 07743 Jena, Germany.; ^6^ European XFEL, Holzkoppel 4, 22869 Schenefeld, Germany.; ^7^ Institut für Quantenoptik, Leibniz Universität Hannover, Welfengarten 1, 30167 Hanover, Germany.; ^8^ Department of Chemistry, Tohoku University, Sendai 980-8578, Japan.; ^9^ Center for Transformative Science and School of Physical Science and Technology, ShanghaiTech University, Shanghai 201210, China.; ^10^ Department of Physics and Astronomy, Louisiana State University, Baton Rouge, LA 70803, USA.

## Abstract

The goal to control short-wavelength radiation for the investigation and manipulation of ultrafast dynamics in quantum systems coevolves with the growing availability of extreme-ultraviolet (XUV) and x-ray sources from high-harmonic generation and free-electron lasers. Here, we present an XUV spatio-spectral phase modulator based on an intense XUV laser beam propagating through an optically thick resonant target, introducing dispersion profile variations around the resonance both perpendicular to and along the laser propagation direction. The resulting dipole radiation gets spectrally reshaped and becomes more divergent as compared to the original beam in the far field. As an experimental demonstration, the intense-XUV–induced double-peak off-axis structure in the far-field spectrum obtained at the Free-Electron Laser in Hamburg (FLASH) shows indications of the underlying XUV-driven Rabi dynamics and resonant pulse propagation effects. The presented work highlights a ubiquitous phenomenon occurring when an intense laser beam passes through a resonant medium.

## Teaser

Intense XUV beams undergo spatial and spectral reshaping during propagation through a resonant medium.

## INTRODUCTION

Ultrashort laser pulses with high intensity have widespread applications in modern society from fundamental research to advanced medical and industrial applications. The interaction of matter with strong laser fields has revealed a wealth of nonlinear phenomena such as ac Stark shift, self-phase modulation, multiphoton ionization, atomic stabilization, and high-harmonic generation (*1*–*5*). The extension of nonlinear spectroscopy from optical to the extreme-ultraviolet (XUV) and x-ray region enables novel insights into electronic dynamics with unprecedented temporal and spatial resolution (*6*, *7*). Recent technical developments in XUV and x-ray free-electron lasers (FELs) at large-scale facilities providing ultrashort and high-brilliance light pulses with tunable wavelengths have opened new research opportunities previously out of reach (*8*–*20*).

The absorption and dispersion of light are omnipresent phenomena in nature. They are connected to the complex refractive index of the interacting medium and can be microscopically described in terms of the induced oscillating electric dipoles. Following the excitation of the system, free induction decay produces reemitted radiation at the resonance frequency (*21*, *22*). By changing the phase and amplitude of the dipole emitters with an external laser field, attosecond transient absorption spectroscopy has enabled the control over the absorption line shapes (*23*–*31*). In the nonperturbative excitation regime, even single-pulse excitation can lead to different absorption profiles for different pulse intensities, through the time-dependent phase shift associated with Rabi cycling between resonant energy levels (*14*, *32*–*36*). Going beyond the dilute-gas limit, the collective light-matter interaction resulting from the macroscopic pulse propagation inside the medium comes into play (*37*, *38*), which gives rise to spectral reshaping near the resonance (*39*–*50*). In addition to altering the absorption properties, an interesting situation arises when the dispersion profile gets modified. Since the wave vector k follows the expression

k(ω,r,z)=∇φ(ω,r,z) (1)

any inhomogeneity in spectral phase φ accumulated due to dispersion along the transverse direction r perpendicular to the incident laser propagation direction z would result in the spatial redirection of the beam. This concept has only been demonstrated in the weak XUV regime by virtue of a spatially offset auxiliary infrared beam (*51*, *52*) or a gas jet with a transverse density gradient (*53*, *54*). However, the demonstration in the nonperturbative XUV regime has not been reported so far, which not only represents a fundamental scenario that demands both theoretical and experimental exploration. With the growing availability of intense XUV and x-ray sources, it also sheds light on interesting directions for future endeavors in shaping short-wavelength radiations, as well as in the application and optimization of x-ray spectroscopy schemes that require dense media (*55*).

**Fig. 1.**
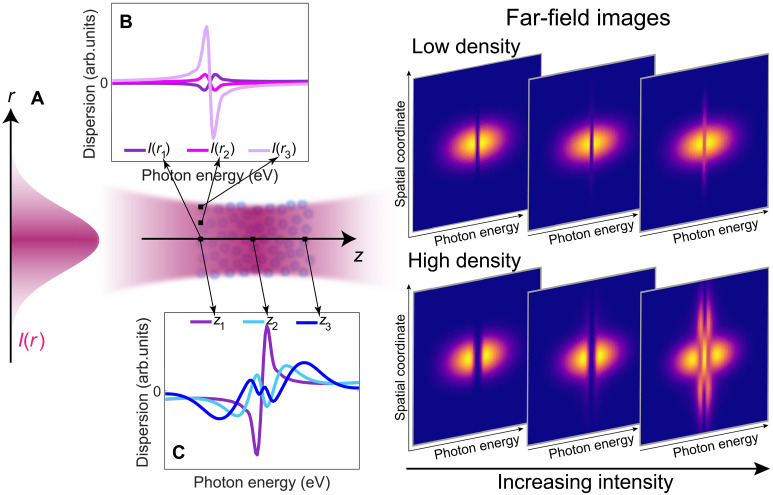
Concept of the XUV spatio-spectral phase modulator. (**A**) A focused XUV beam (cylindrical symmetry is assumed) passes through a target medium and imprints a spectral hole at the transition energy in the transmitted spatio-spectral profile (spatial coordinate: *x* or *y* in Cartesian coordinate). When the pulse intensity is high enough, the refractive index (dispersion) of the target in the vicinity of the resonance undergoes self-induced modification due to the nonlinear interaction, as discussed in (*14*, *32*–*35*). This modification of the spectral phase of the laser depends on the pulse intensity and hence varies along the transverse direction (**B**). The medium thus imparts a nonuniform phase shift to the XUV beam, thereby causing wavefront curvature and redirection of the resonant spectrum. For a medium with a higher target density, macroscopic pulse propagation manifests itself in further modification of the dispersion profiles (**C**) and the reshaping of the resonant spectral structures. The dispersion profiles in (B) and (C) are shown in arbitrary units for better visibility.

In this work, we demonstrate the spatial redirection and spectral reshaping of intense XUV radiation by passing solely a single XUV beam through a resonant medium. This is achieved by the self-induced change of the dispersion profile in the vicinity of an atomic resonance through the interplay of nonlinear light-matter interaction and resonant pulse propagation, thus modifying the spectral phase accumulation in Eq. 1 into φ[ω,r,I (r),z] and inducing a time-dependent lens that deflects the beam. The schematic principle of the concept is presented in Fig. 1. A focused XUV beam propagates through the medium, which acts as a spatio-spectral phase modulator. When the interaction is in the nonperturbative regime, a frequency-dependent modification of the refractive index around the resonance is introduced, which is intensity-dependent and hence varies along the radial direction as shown in Fig. 1B, leading to more divergent radiation close to the resonance as compared to the original beam in the far field. For a medium with a higher target density, the refractive index gets further distorted along the propagation direction (Fig. 1C) as the driving pulse undergoes temporal reshaping, giving rise to the appearance of more spectral structures near the resonance position.

## RESULTS

We start with a simple computational model to identify the characteristic features of the spatial redirection and spectral reshaping of an intense laser beam propagating through macroscopic media. The considered model system consists of the ground state $1s^{2}$ and the singly excited state 1s2p of helium, and is subjected to an ultrashort XUV pulse. The dynamical evolution of the system and the spatial evolution of the XUV spectrum are obtained by solving the coupled time-dependent Schrödinger equation (TDSE) and a one-dimensional propagation equation. More details on the model can be found in “Model calculation” section in Materials and Methods.

**Fig. 2.**
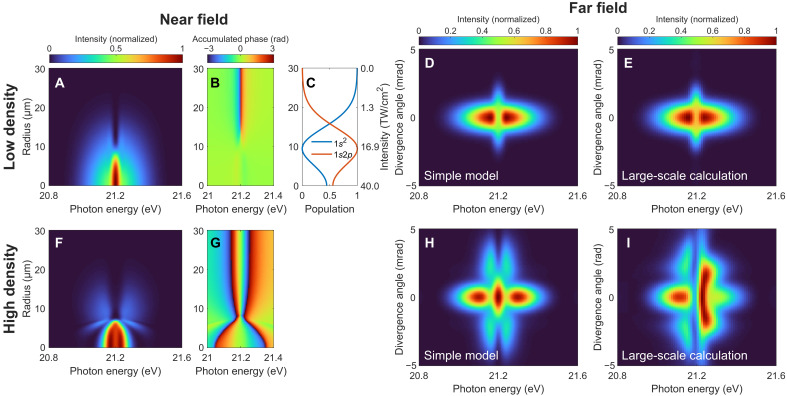
Model simulation of the intense-XUV–induced spatial redirection and spectral reshaping in optically thin and thick media. (**A** and **F**) Near-field radial-spectral profile, (**B** and **G**) accumulated phase of the XUV beam at the exit of the medium, and the resulting (**D** and **H**) far-field spatio-spectral profiles. (**C**) Evolution of the state populations after the interaction with the incoming pulse as a function of radial distance from the beam center (i.e., peak XUV pulse intensity) calculated from the two-level model. (**E** and **I**) Far-field spatio-spectral profiles obtained from the large-scale TDSE-MWE simulations. The atomic densities in (A) to (E) and (F) to (I) are 2.4 × 10^16^ and 1.9 × 10^17^ cm^−3^, respectively. The evolution of the state populations in the high-density case depends also on the propagation distance *z* as the driving XUV pulse undergoes temporal reshaping, and (C) essentially represents the results at the entrance of the medium.

We choose a cosine-squared XUV pulse of 8-fs full width at half maximum duration, centered on resonance at 21.2 eV, with a peak intensity of 40 TW/cm^2^. The transform-limited bandwidth of 0.2 eV is comparable to the measured averaged FEL bandwidth in the experiment shown below, and its pulse duration represents a single temporal SASE (self-amplified spontaneous emission) spike contained within the FEL pulse. This treatment captures coherent coupling effects and is sufficient to qualitatively reproduce the experimental findings, as demonstrated in previous works (*14*, *56*, *57*). The dipole matrix element between the two states is 0.3 atomic units. We consider a constant Gaussian beam radius of 15 μm propagating through the target medium of 2 mm in length. A window function of 110-fs width is imposed on the dipole evolution in the time domain to account for the dephasing effect. The calculated radial-spectral profile of the XUV beam at the exit of the medium is shown in Fig. 2A, for a rather dilute medium with an atomic number density of 2.4 × 10^16^ cm^−3^, where the pulse reshaping effect is insignificant and the results are similar to the single-atom prediction. With decreasing radial distance toward the center of the beam, corresponding to increasing peak pulse intensity, the spectral profile in the vicinity of the resonance in the transmitted spectrum changes from a dip to a peak. This spectral lineshape change is accompanied by a spectral phase change as shown in Fig. 2B and is associated with the substantial population transfer between the states as shown in Fig. 2C. For the resonant case considered here, this phase jump is related to the sign change of the ground-state coefficient when the pulse area exceeds π (*35*, *58*). It indicates that the electrons returning to the ground state after completing half a Rabi cycle carry an additional phase of π, thus turning the natural absorption of the resonance into emission. The Rabi oscillations driven by intense short-wavelength light has received considerable interest in recent years (*14*, *17*, *20*, *59*) and is a signature of the nonlinear XUV light-matter interaction. The resulting spatial phase variation along the radial axis leads to a redirected XUV emission in the far field at the resonant frequency, which is plotted in Fig. 2D. The above analysis sets a criterion for the observation of spatial redirection in the resonant case: The peak laser intensity needs to be higher than a certain threshold, such that the pulse area exceeds a value of π, which is ∼18 TW/cm^2^ in the presented case. For higher atomic densities, the collective light-matter interaction within the medium leads to strong temporal and spectral pulse reshaping (*39*–*41*). Since a phase change of more than π in the frequency domain will lead to a sign change and thereby the formation of a new subpulse in the time domain (i.e., substantial temporal reshaping), propagation effects become important when the accumulated spectral phase is larger than π (*40*), which corresponds to a density higher than ∼7 × 10^16^ cm^−3^ in the presented case. For a considered atomic density of 1.9 × 10^17^ cm^−3^, the peak in the near-field spectrum develops into two as presented in Fig. 2F, accompanied by a broadened spectral phase profile as shown in Fig. 2G, which translates into two off-axis emission peaks in the far field in Fig. 2H. When the driving pulse intensity is not sufficient to induce a spectral phase variation along the radial direction, no spatial redirection occurs for both the low- and high-density cases, as shown in the “Simulation results for the low-intensity case” section. We can thus identify the XUV light-matter interaction at high intensity as a key ingredient to observe a nontrivial off-axis emission pattern.

To demonstrate the robustness of the effect, we proceed with a large-scale calculation to go beyond the few-level description of the system and the uncoupled radial-spectral evolution of the XUV beam. The calculation is performed by solving the three-dimensional (3D) coupled TDSE in the single-active-electron (SAE) approximation and the Maxwell wave equation (MWE) in helium gas (*42*). The same XUV laser and medium parameters are used as before except for an XUV central photon energy of 21.1 eV, which is used to meet the resonant condition as the applied pseudopotential in the SAE-TDSE calculation yields a 1s2p state energy of 21.1 eV (instead of 21.2 eV measured experimentally and used in the simple model). Moreover, a confocal parameter of 2.5 cm is used, which corresponds to a Gaussian beam waist of 15 μm. The calculated spatio-spectral profiles in the far field for the two atomic densities are shown in Fig. 2 (E and I), respectively. A 0.1-eV shift of the energy axis is implemented to accommodate the energy offset from the preceding value. The result in the low-density case shown in Fig. 2E agrees well with the simple model simulation in Fig. 2D, in terms of the single off-axis emission peak at the resonant frequency. However, the spectral profile becomes asymmetric in Fig. 2I as compared to its counterpart from the simple model simulation in Fig. 2H. We confirm that this difference between them predominantly originates from the wavefront curvature of the focused beam: A larger XUV confocal parameter in the 3D TDSE-MWE calculation leads to less asymmetric profiles, which agree more with the simple model prediction in which the focusing and defocusing of the XUV beam within the medium are neglected. It represents a breakdown of the simplest uncoupled approximation for the radial phase variation in the high-density case and indicates the spatio-spectral coupling (*60*) introduced during the realistic interaction scenario, which merits detailed future exploration. Nevertheless, Fig. 2 (H and I) shows clear double-peak structure in the off-axis spatio-spectral profile, which serves as a hallmark for later experimental demonstration of the interplay between intense XUV light-matter interaction and resonant pulse propagation in an optically thick medium.

**Fig. 3.**
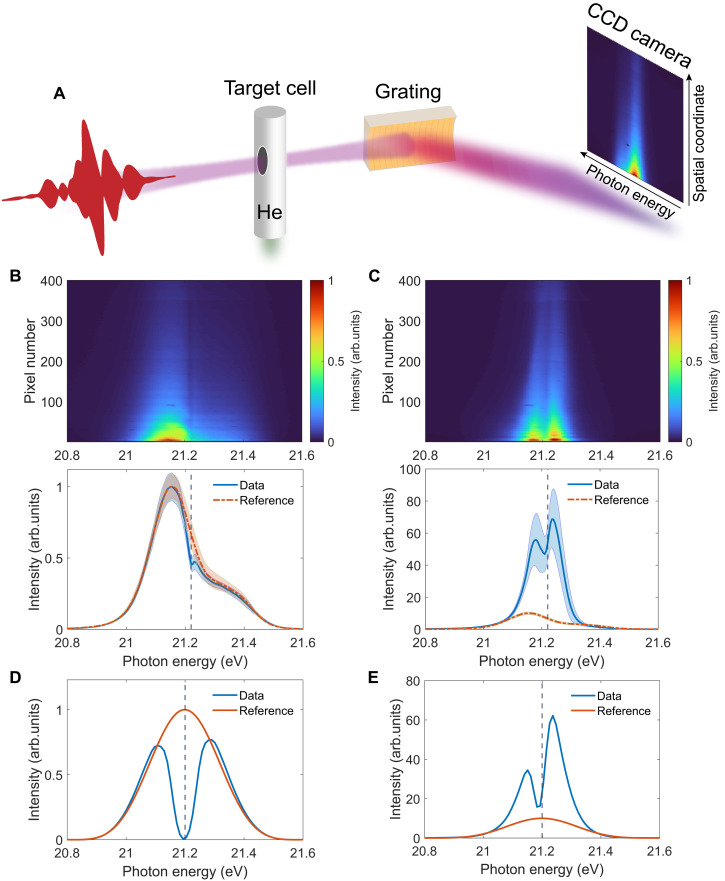
Experimental signature of the intense-XUV–induced spatial redirection and spectral reshaping. (**A**) Sketch of the experimental setup. The main on-axis part of the dispersed XUV FEL beam was underneath the CCD camera, and only the far off-axis part was experimentally recorded due to a fixed experimental geometry during the beamtime. Recorded 2D CCD image (top) and the spectrum integrated over the detected off-axis spatial range (bottom, blue solid line) for the (**B**) attenuated and (**C**) high-intensity FEL beam. Note that the lowest pixel on the vertical axis does not represent a point on the optical axis but some finite distance from the optical axis. The vertical gray dashed line marks the energy position of the 1s2p resonance. The orange dash-dotted line shows the normalized reference spectrum (see main text). The shaded areas indicate the corresponding SD of the measurement (see the Supplementary Materials). Simulated far-field off-axis spectra for the (**D**) low- and (**E**) high-intensity cases. The spectra are spatially integrated for the divergence range of 1.1 to 5 mrad in Figs. 4H and 2I, respectively. The reference spectra are obtained in the absence of target gas. The simulation in (D) overestimates the absorption because of the artificially shortened dephasing time (see main text). The key experimental features shown in the bottom panels of (B) and (C) are well captured.

Now, we present an experimental signature of the described mechanism by observing the intense-FEL–induced double-peak structure in the transmitted off-axis XUV spectrum. The experiment was performed with the SASE FEL pulses at FLASH, DESY (*61*), and the experimental setup is sketched in Fig. 3A. More details on the experiment can be found in the “Experiment” section in Materials and Methods. Figure 3B shows the measured spatio-spectral profile on the 2D charge-coupled device (CCD) camera and the spatially integrated spectrum for an attenuated FEL beam with a central photon energy of 21.2 eV. The nominal backing pressure of the gas cell was fixed at around 26 mbar, which corresponds to an atomic number density of 6.3 × 10^17^ cm^−3^ for an ideal gas at room temperature. However, we note that the actual pressure in the interaction region is nonuniform and generally smaller than the nominal backing pressure. An absorption dip at the resonant position is observed, which agrees with the simulation results in Fig. 3D. We would like to note that the faint absorption dip observed in the experiment is due to the long natural lifetime of the 1s2p state of 0.57 ns (*62*) (∼1-μeV natural linewidth), whose spectral feature gets substantially blurred by the finite spectrometer resolution of ∼30 meV (*63*), while an artificially shortened dephasing time is used in the simulation to reduce the computational cost, resulting in overestimated absorption as shown in Fig. 3D. The reference spectrum in the experiment is recorded with a central FEL photon energy of ∼22.1 eV, at which the helium gas is resonance-free and essentially transparent to the incoming XUV FEL beam. The reference spectrum is shifted in photon energy to 21.2 eV in Fig. 3 (B and C), and its close overlap with the signal outside the resonant region in Fig. 3B reflects the robustness of the FEL spectrum. For the results of the high-intensity FEL beam shown in Fig. 3C, an obvious double-peak structure around the resonance position appears, with an amplitude considerably larger than that of the reference FEL spectrum. This double-peak feature is considerably broader than the absorption profile but narrower than the driving FEL spectrum, in agreement with the simulation results shown in Fig. 3E. Note that the measured two off-axis emission peaks are asymmetric, which is a signature of the spatio-spectral coupling predicted by the large-scale TDSE-MWE calculation. These intensity-dependent spectral behaviors mirror the theoretical simulations in the high-density case, which confirm the experimental observation of the spatial redirection and spectral reshaping of intense XUV radiation. Hereby, the characteristic double-peak off-axis structure observed in Fig. 3C signifies the entry into the high-XUV intensity and high-pressure regime, in which coupled XUV-driven Rabi dynamics and resonant pulse propagation effects are at work.

### Simulation results for the low-intensity case

**Fig. 4.**
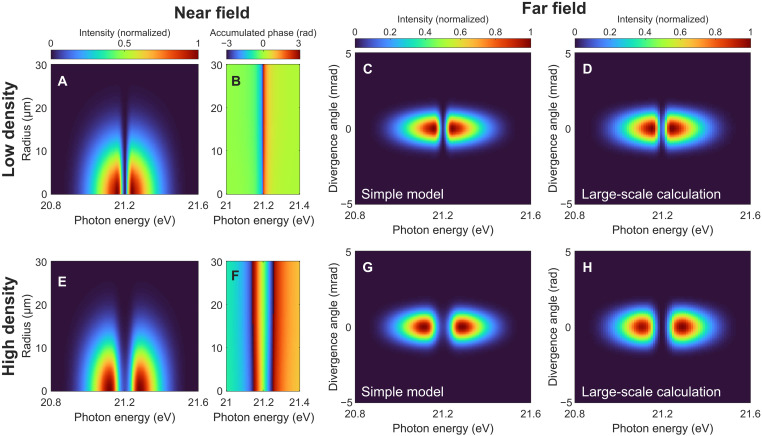
Simulation results for the low-intensity case. (**A** and **E**) Near-field radial-spectral profile, (**B** and **F**) accumulated phase of the XUV beam at the exit of the medium, and the resulting (**C** and **G**) far-field spatio-spectral profiles simulated with the same parameters as in Fig. 2 except for a peak laser intensity of 4 TW/cm^2^. (**D** and **H**) Far-field spatio-spectral profiles obtained from the large-scale TDSE-MWE simulations. The atomic densities in (A) to (D) and (E) to (H) are 2.4 × 10^16^ and 1.9 × 10^17^ cm^−3^, respectively.

Figure 4 presents the simulated near- and far-field results with the same parameters as in Fig. 2 except for a rather low peak XUV pulse intensity of 4 TW/cm^2^. Here, the accumulated spectral phase of the XUV beam is uniform along the radial direction for both the low- (Fig. 4B) and high-density (Fig. 4F) cases. A spectral dip appears at the transition energy in the far-field spectrum (Fig. 4, C, D, G, and H), and no spatial redirection of the XUV beam occurs.

### Simulation results for the blue-detuned case

**Fig. 5.**
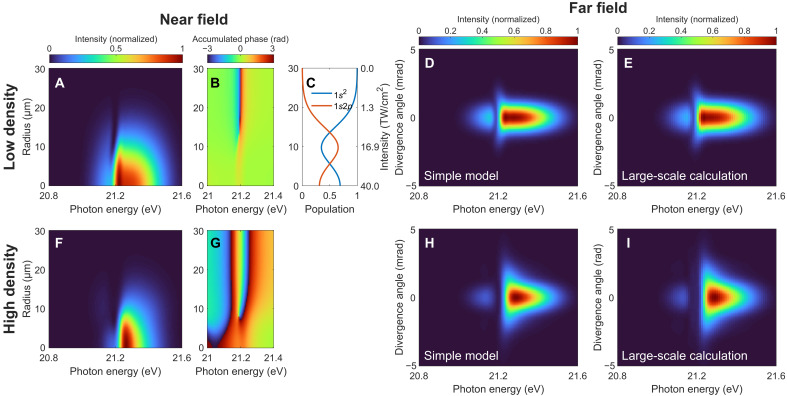
Same simulation as shown in Fig. 2, except for a blue detuning of 0.1 eV. (**A** and **F**) Near-field radial-spectral profile, (**B** and **G**) accumulated phase of the XUV beam at the exit of the medium, and the resulting (**D** and **H**) far-field spatio-spectral profiles. (**C**) Evolution of the state populations after the interaction with the incoming pulse as a function of radial distance from the beam center (i.e., peak XUV pulse intensity) calculated from the two-level model. (**E** and **I**) Far-field spatio-spectral profiles obtained from the large-scale TDSE-MWE simulations. The atomic densities in (A) to (E) and (F) to (I) are 2.4 × 10^16^ and 1.9 × 10^17^ cm^−3^, respectively.

Figure 5 presents the same simulation results as shown in Fig. 2, except for a blue detuning of 0.1 eV. In contrast to the resonant case, the spectral lineshape and the accumulated phase change continuously with radial distance from the beam center (i.e., XUV peak intensity) as shown in Fig. 5, A and B, indicating a reduced requirement of pulse intensity for the observation of the spatial beam redirection. In addition, a dominant off-axis peak appears with a photon energy higher than the resonance in Fig. 5 (H and I). These results agree qualitatively with the experimental observations for the blue-detuned case in fig. S4 in the Supplementary Materials.

## DISCUSSION

While the observed set of two peaks for the resonant case is easily reminiscent of the Autler-Townes doublet (*64*), it is not a direct consequence of the Autler-Townes effect as no spectral splitting appears in the low-density case in Fig. 2. The Autler-Townes effect describes the transient energy splitting of a two-level system subjected to a strong driving pulse, with the splitting being given by the generalized Rabi frequency. If the transition is probed from a third state during the interaction [e.g., by photoionization as shown in Refs. (*17*, *20*)], then the splitting of the energy levels can be measured. For the amplified spontaneous emission of a short-lived resonance after core ionization, self-induced Autler-Townes splitting of the stimulated emission spectra is observed when reaching the saturation regime both theoretically (*65*) and experimentally (*57*). There, an initially excited system is strongly coupled by the emitted light, whereas in our and more general cases, one starts from a system with all population in the ground state. When the induced response of the system is limited to the driving pulse duration, the Mollow-like triplet patterns in the resonant absorption of a strongly driven system can form under certain conditions (*66*). Compared with the response induced within the driving pulse duration, the free-induction decay of a long-lived resonance lasts longer in time and contributes more to the transmitted laser spectrum. In the presented case, the spacing between the two off-axis peaks depends on the pathlength-density product (see more calculation results in the Supplementary Materials), as the underlying mechanism is a macroscopic propagation effect different in nature from the above mentioned works.

In summary, we have demonstrated the modification of the spatial and spectral properties of XUV radiation by strongly driving an electronic transition with an intense XUV FEL beam, which translates the radial intensity inhomogeneity into different spectral phase variations in the vicinity of the resonance. The interplay between intense XUV light-matter interaction and resonant pulse propagation in an optically thick medium leads to the redirection and reshaping of the spectral structures. In combination with theory calculations, the characteristic two off-axis emission peaks in the resonant case indicate the presence of XUV-driven Rabi dynamics and macroscopic pulse propagation effects. The slightly detuned driving pulse relaxes the requirement of pulse intensity for the observation of the spatial beam redirection. We expect our work to stimulate future experimental activities on this concept with more laser beam and medium parameters, as well as the simultaneous detection of the on-axis spectrum to provide a comprehensive and more detailed description of the reported effect. In addition, using XUV sources with fewer temporal structures, such as seeded or single-spike FELs or intense pulses recently produced from high-harmonic generation (*67*), can help to better study and control the underlying dynamics and the resulting spatial and spectral reshaping. Our work represents a substantial advance in understanding the propagation of intense XUV light near electronic transitions, a fundamental light-matter interaction process that is lacking description thus far. The application of novel spectroscopic techniques such as stimulated x-ray Raman spectroscopy (*55*, *68*) would also benefit from a solid understanding of the nonlinear interaction of resonant high-frequency light with matter. As a resonant effect, the uncovered mechanism is expected to be generally applicable, including atom-specific core transitions in the x-ray domain. The described spatio-spectral phase modulators working in the intense short-wavelength regime could be potentially applied to enhance the spatial and spectral coherence properties of pulses with initially statistical spectral shapes by passing them near resonances for nonlinear filtering, or even provide a new method of nonlinear beam guiding.

## MATERIALS AND METHODS

### Model calculation

The wave function of the system can be written as $|\Psi \left(t\right)\rangle =c_{1}\left(t\right)|1s^{2}\rangle +c_{2}\left(t\right)e^{-i\omega_{12}t}|1s2p\rangle$, with $\omega_{12}$ denoting the transition frequency. The evolution of the state coefficients $c_{1}\left(t\right)$ and $c_{2}\left(t\right)$ when the system is subjected to an ultrashort XUV pulse E(t) is computed by solving the TDSE. The spatial evolution of the XUV spectrum in one dimension along the propagation direction is approximated by (*69*)

$$\frac{\partial }{\partial z}\tilde{E}\left(\omega ,z\right)=-2\pi i\frac{\omega }{c}\tilde{P}\left(\omega ,z\right)$$ (2)

which was formulated in a reference frame moving with the XUV laser pulse at the speed of light in vacuum c. The polarization response in the frequency domain $\tilde{P}\left(\omega ,z\right)$ is related to the dipole spectrum $\tilde{d}\left(\omega ,z\right)$ by $\tilde{P}\left(\omega ,z\right)=2N\tilde{d}\left(\omega ,z\right)$, in which *N* denotes the atomic number density and the factor 2 accounts for the response of two electrons in helium. Here, we decouple the XUV radial and spectral evolution to isolate the effect induced by different pulse intensities: The focusing and defocusing of the XUV beam within the medium is neglected, and each point along the radial direction is treated independently with different input peak intensities following a Gaussian distribution. In this case, any induced inhomogeneity in spectral phase φ along the radial direction in Eq. 1 originates from the nonuniform radial intensity distribution. To convert the transmitted XUV spectrum from the near field to the far field where it is actually recorded in the experiment, a Hankel transformation is further performed to account for the propagation of the XUV beam in free space (*70*).

### Experiment

As illustrated in Fig. 3A, the FEL beam was focused into a helium-filled gas cell with an interaction length of 2 mm. The transmitted beam was dispersed by an aberration-corrected concave grating (Hitachi, 001-0639) with a variable line spacing (*71*) and detected in the far field by a 2D CCD camera (Princeton Instruments, PIXIS-XO: 400B), which records the 2D XUV spatio-spectral profile with one spatial axis and one spectral axis. The grating focuses the FEL beam along the horizontal plane on the camera chip, such that the horizontal axis on the camera is truly the dispersive energy-resolving axis, while the angular divergence of the beam is reflected in the spatial distribution along the vertical axis. The design and geometry of the grating have been optimized to minimize aberrations on the detector plane (*72*), and an analysis of the spectral imaging properties is presented in the Supplementary Materials. We note that in the experimental configuration, the main on-axis part of the beam was underneath the CCD camera, and only the far off-axis FEL beam profile was recorded. We are thus more sensitive to the change of the off-axis XUV spectra since the CCD camera has a limited dynamical range, which leads to the presented experimental observations, albeit the on-axis information is lost. Obtaining the full far-field profile would provide more information on the processes under study but was not available due to a fixed experimental geometry during the beamtime. The FEL was operated in single-bunch mode at 10-Hz repetition rate with a pulse energy of ∼15 μJ. The bandwidth of the averaged spectrum was measured to be ∼0.2 eV. Considering the beamline transmission of ∼30%, the estimated average SASE pulse duration of ∼70 to 90 fs, and the focal beam size of about 10 μm, the average on-target peak pulse intensity was in the 10^13^ W/cm^2^ range, which is beyond the perturbative regime (*73*). An aluminum filter of 400-nm thickness can be inserted into the beam path to attenuate the incoming beam. Taking into account an aluminum oxide layer (a few nanometer thickness) on the filter surfaces, we estimate an order of magnitude of attenuation in the considered photon energy range of around 21 eV based on the metal filter transmission curves (*74*), which is close to the perturbative regime (*73*).
