# Supplementary material for: Redirection and reshaping of intense extreme-ultraviolet radiation
Source: Sci Adv. 2026 May 29;12(22):eaef5300. doi: 10.1126/sciadv.aef5300 (PMC13220865; doi:10.1126/sciadv.aef5300)
Supplement: Supplementary file 1 — Supplementary Text Figs. S1 to S8 [file sciadv.aef5300_sm.pdf]

Science Advances
AAAS

Supplementary Materials for

Redirection and reshaping of intense extreme-ultraviolet radiation

Yu He *et al.*

Corresponding author: Yu He, yuhe@mpi-hd.mpg.de; Christian Ott, christian.ott@mpi-hd.mpg.de; Thomas Pfeifer, thomas.pfeifer@mpi-hd.mpg.de

Sci. Adv. **12**, eaef5300 (2026)
DOI: 10.1126/sciadv.aef5300

This PDF file includes:

Supplementary Text
Figs. S1 to S8

I. Reproducibility of the SASE FEL spectrum

Figure S1(a) shows the single-shot, non-spatially resolved SASE FEL spectra recorded with a central photon energy of $\sim$20.1 eV. The considerable overlap between the averaged spectrum and the reference spectrum shown in Fig. S1(b), along with the results presented in the lower panel of Fig. 3B in the main text, shows the reproducibility of the averaged FEL spectrum over the course of the measurements.

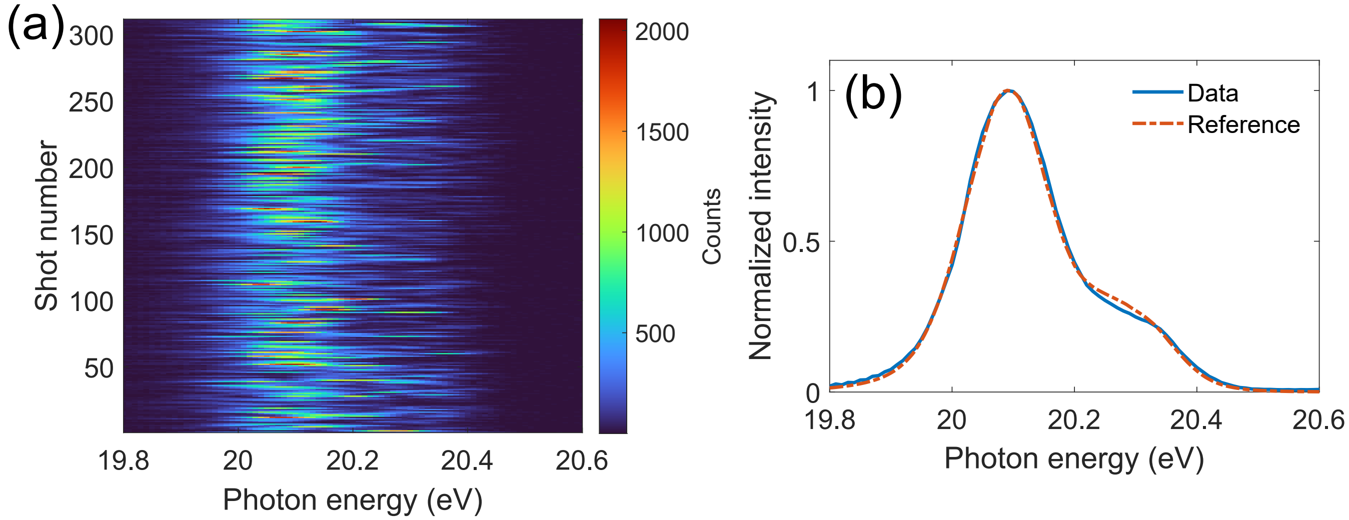

FIG. S1. **Reproducibility of the SASE FEL spectrum.** (a) Single-shot, non-spatially resolved SASE FEL spectra recorded with a central photon energy of $\sim$20.1 eV. (b) Averaged spectrum (normalized, blue line) and the normalized reference spectrum [orange dash-dotted line, shifted in energy, see Fig. S2(b)] with a central photon energy of $\sim$22.1 eV recorded 1 hour later in time.

II. Statistics of the measured results

The statistics of the measured results shown in the main manuscript are presented in Fig. S2. The left panels [Figs. S2(a), S2(c) and S2(e)] show the individual spectra recorded each with 100 FEL shots, and the averaged spectra and the associated standard deviation are shown in the right panels [Figs. S2(b), S2(d) and S2(f)]. The presence of the two emission peaks in the high-intensity case in Fig. S2(e) is persistent.

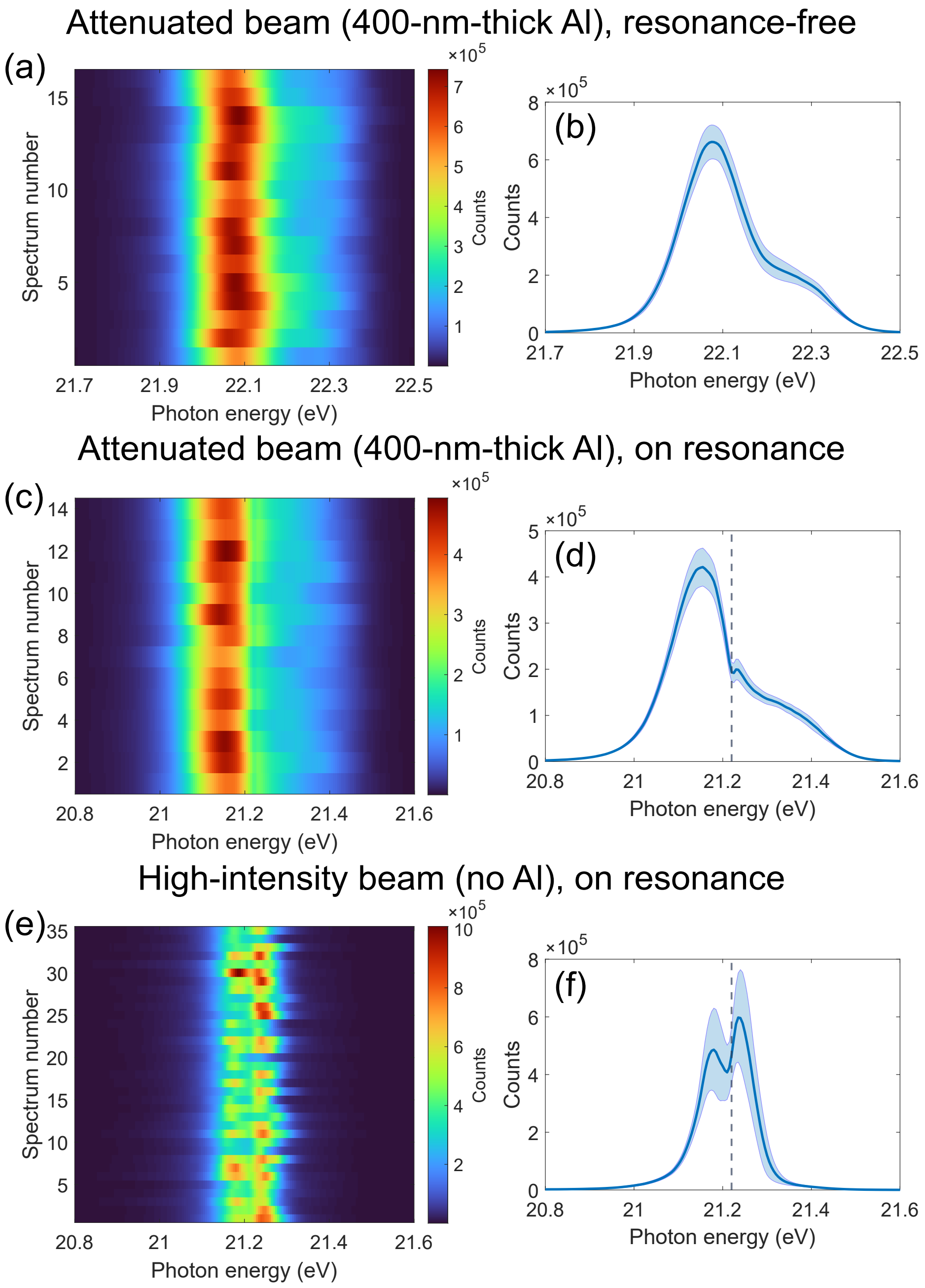

FIG. S2. **Statistics of the measured results for the resonant case.** (a, c, e) Individual spectra integrated over the whole spatial range of the CCD camera chip, each recorded with 100 SASE FEL shots. (b, d, f) Averaged spectra and the associated standard deviation (blue shaded area). The vertical gray dashed line marks the energy position of the $1s2p$ resonance of helium. We note that for the case of high-intensity FEL beam in (e) and (f), a 500-nm-thick aluminum filter was placed downstream of the target cell to reduce the counts on the CCD camera to avoid saturation.

III. Experimental results at intermediate intensities for the resonant case

Figure S3 shows the additional experimental results for the resonant case at two intermediate intensities, at which the incoming FEL beam was attenuated by an Al filter of 200- and 100-nm thickness, which corresponds to a transmission rate of $\sim$36% and $\sim$46%, respectively. For the case with a 200-nm-thick Al filter [Figs. S3(a) and S3(b)], the attenuation of the spectral intensity around the resonance is robust. When the 100-nm-thick Al filter is used [Figs. S3(c) and S3(d)], only a few spectra show significant increase in counts, which shall be connected to the intrinsic intensity fluctuation of the SASE FEL beam. It supports the criterion presented in the main text that a threshold intensity needs to be reached for the observation of spatial redirection for the resonant case.

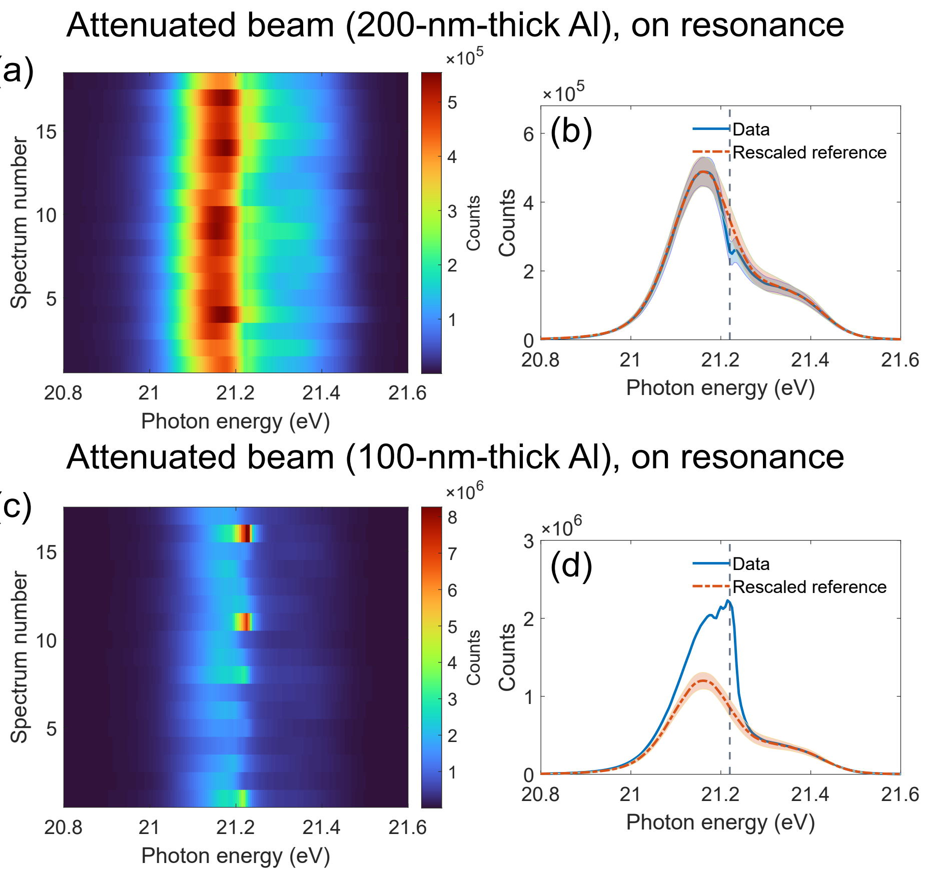

FIG. S3. **Experimental results recorded at intermediate intensities for the resonant case.** (a, c) Individual spectra integrated over the whole spatial range of the CCD camera chip, each recorded with 100 SASE FEL shots. (b, d) Averaged spectra and the associated standard deviation (blue shaded area). The vertical gray dashed line marks the energy position of the $1s2p$ resonance of helium. The reference spectra in (b) and (d) are rescaled to match the wings of the signal. As the data of the blue curve in (d) is highly right-skewed, the standard deviation is not suitable for showing the statistics.

IV. Experimental results for the blue-detuned case

Figure S4 shows the measured experimental results for the blue-detuned case at which the FEL central photon energy is tuned to 21.3 eV. Compared to the reference signal, the attenuation of the spectrum around the resonance is observed for the lower-intensity cases as shown in Figs. S4(b) and S4(d). In contrast to the double-peak structure for the resonant case, a dominant peak with a higher photon energy than the resonance appears for the higher-intensity cases as shown in Figs. S4(f) and S4(h).

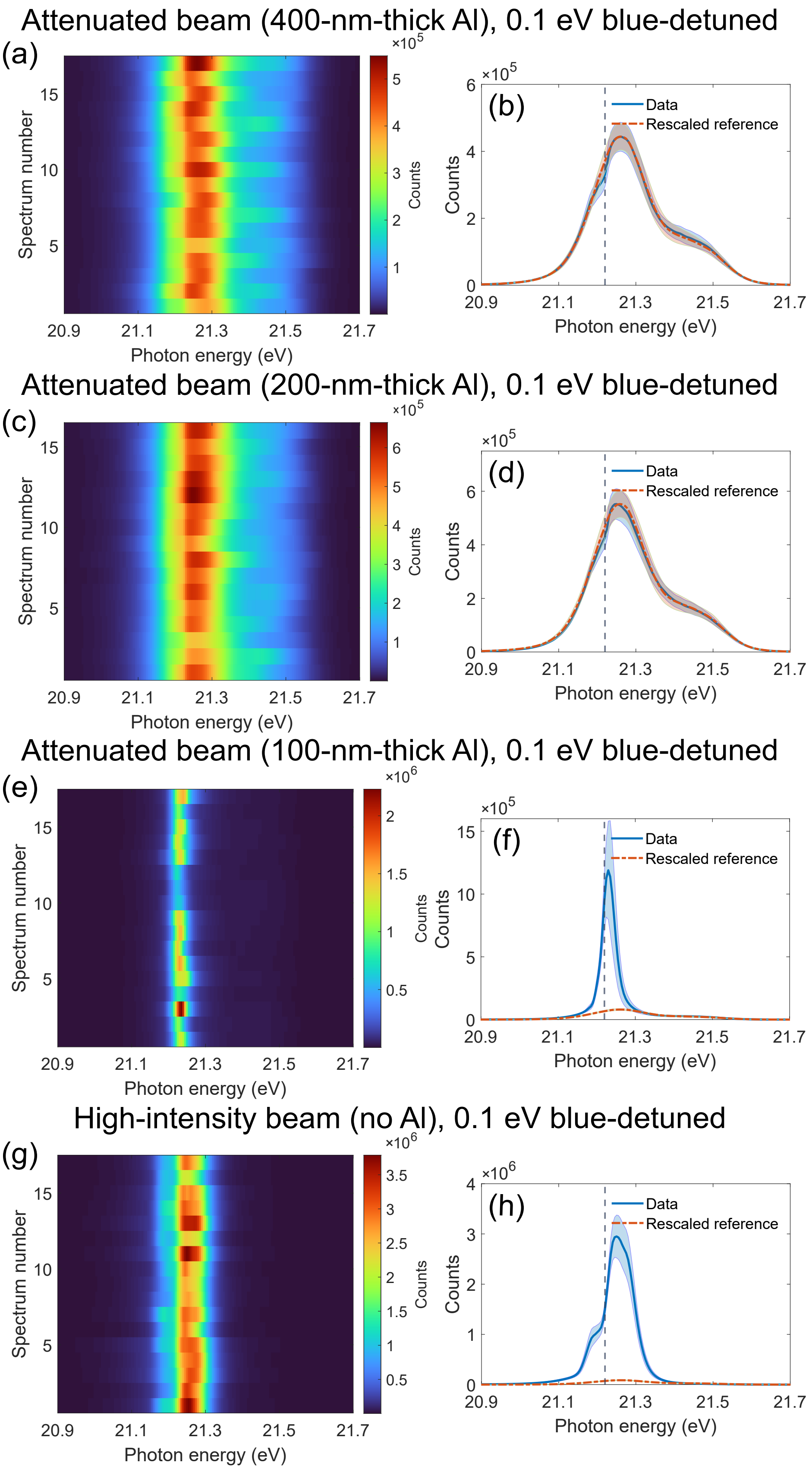

FIG. S4. **Experimental results for the blue-detuned case.** (a, c, e, g) Individual spectra integrated over the whole spatial range of the CCD camera chip, each recorded with 100 SASE FEL shots. (b, d, f, h) Averaged spectra and the associated standard deviation (blue shaded area). The vertical gray dashed line marks the energy position of the $1s2p$ resonance of helium. Note that to avoid saturation, aluminum filters with thicknesses of (e, f) 200 and (g, h) 500 nm were placed downstream of the target cell to reduce the counts on the CCD camera, respectively.

V. Additional simulation results for the resonant case

Additional simulation results are presented in this section to reveal the influence of atomic density, peak pulse intensity, and different combinations of target density and medium length for the resonant case. The other laser and medium parameters are the same as those used in Fig. 2I in the main text.

Figure S5 presents the far-field spatio-spectral profiles obtained from the large-scale TDSE-MWE simulations for three different atomic densities. At the intermediate density of $9.5 \times 16\ \mathrm{cm}^{-3}$ shown in Fig. S5(a), the off-axis double-peak structure around the resonance already appears, while the splitting is less significant as compared to the experimental results in Fig. 3C in the main text. For even higher atomic densities shown in Figs. S5(b) and S5(c), the off-axis feature becomes more divergent with more structures appearing.

The simulated far-field spatio-spectral profiles for three different peak intensities are shown in Fig. S6. Redirection of the XUV spectrum appears at peak intensities of 20 [Fig. S6(b)] and 80 $\mathrm{TW/cm}^2$ [Fig. S6(c)], but is absent even for a moderate peak intensity of 10 $\mathrm{TW/cm}^2$ [Fig. S6(a)]. Note that the pulse area for a peak intensity of 80 $\mathrm{TW/cm}^2$ is more than 2π (more than one full Rabi cycle) and the discussion of this case is beyond the scope of the present work.

The simulation results for three different combinations of target density and medium length, but keeping their product constant, are shown in Fig. S7. The overall profiles agree well with each other, with only slight differences due to the 3D focused beam geometry and the coupled radial-spectral evolution.

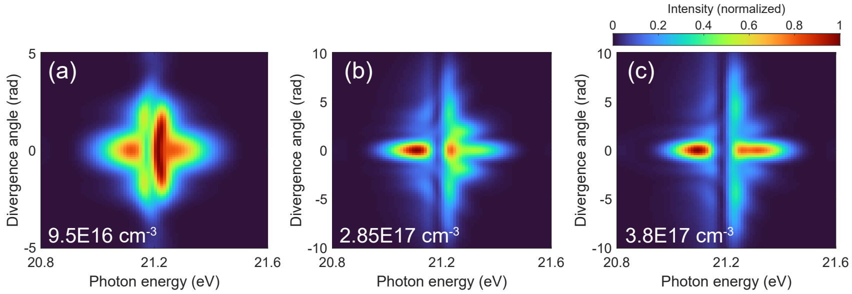

FIG. S5. **Far-field spatio-spectral profiles obtained from the large-scale TDSE-MWE simulations for three different atomic densities.** (a) $9.5 \times 16\ \mathrm{cm}^{-3}$. (b) $2.85 \times 17\ \mathrm{cm}^{-3}$. (c) $3.8 \times 17\ \mathrm{cm}^{-3}$.

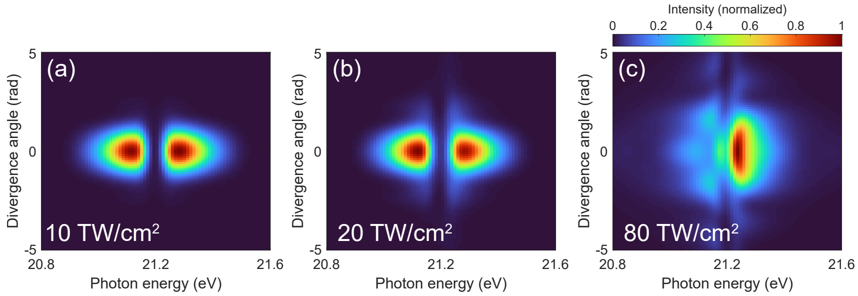

FIG. S6. **Far-field spatio-spectral profiles obtained from the large-scale TDSE-MWE simulations for three different peak intensities.** (a) 10 $\mathrm{TW/cm}^2$. (b) 20 $\mathrm{TW/cm}^2$. (c) 80 $\mathrm{TW/cm}^2$.

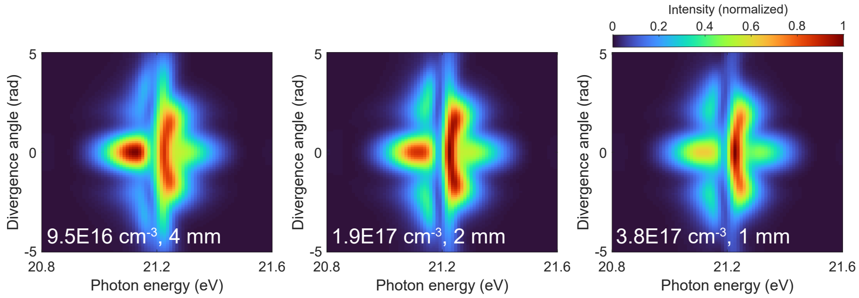

FIG. S7. **Far-field spatio-spectral profiles obtained from the large-scale TDSE-MWE simulations for three different combinations of target density and medium length, but keeping their product constant.** (a) $9.5 \times 16\ \mathrm{cm}^{-3}$, 4 mm. (b) $1.9 \times 17\ \mathrm{cm}^{-3}$, 2 mm. (c) $3.8 \times 17\ \mathrm{cm}^{-3}$, 1 mm.

VI. Analysis of the spectral imaging properties of the concave grating with variable-line-space grooves

Figure S8(a) shows the schematic layout of the concave grating spectrometer. To analyze the spectral imaging (focusing) property, we adopt a rectangular coordinate system and neglect the vertical direction here. The origin of the coordinate O is at the vertex of the concave grating sphere. The X axis is normal to the concave surface at O, and the Y axis is perpendicular to the grating groove at O. The groove spacing σ is defined as a function of groove position y

$$\sigma(y) = \sigma_0/(1 + \frac{2b_2}{R}y + \frac{3b_3}{R^2}y^2 + \frac{4b_4}{R^3}y^3 + ...), \tag{1}$$

where σ_0 is the groove spacing at O, R is the radius of curvature of the grating, and b_2, b_3, b_4, ... are the parameters for space variation.

We consider the extreme case that the whole horizontal surface of the grating is fully illuminated by the light from the entrance slit. Our laser focus at the interaction region serves as a virtual slit, and it is treated as a point object because of its small dimension. The intersection of the light diffracted at the horizontal edges on the grating surface $\mathrm{C}_1(R - \sqrt{R^2 - W^2/4}, W/2)$ and $\mathrm{C}_2(R - \sqrt{R^2 - W^2/4}, -W/2)$ is regarded as the focus, where W is the width of the grating surface. Following the grating equation, the calculated focal curve of the grating used in the experiment (Hitachi 001-0639) for the wavelength range between 22 nm (56.36 eV) and 124 nm (10 eV) is shown in Fig. S8(b), which is indeed in close proximity to the given spectral plane at $Y = -d_2 = -469$ mm.

The calculated incident and outgoing light at 21.2 eV (58.48 nm) diffracted on the entire grating surface is shown in Fig. S8(c). We find that in the given geometry, the spread of the diffracted light on the spectral plane is within 15 μm. It is smaller than the used pixel size of the CCD camera of 20 µm, thus the aberration on the detector plane is inconsiderable for the present spectrometer even when the entire horizontal surface of the grating is illuminated.

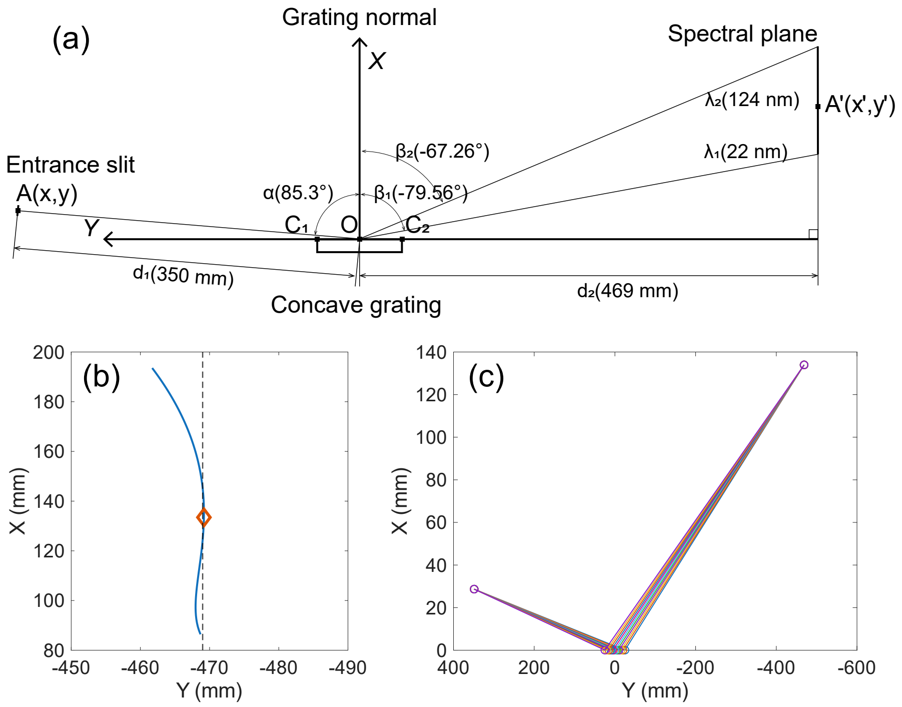

FIG. S8. **Spectral imaging properties of the grating.** (a) Schematic diagram and design specifications for the flat-field grazing-incidence spectrograph using a mechanically ruled aberration-corrected concave grating (Hitachi 001-0639). Nominal groove spacing $\sigma_0 = 1/600$ mm; radius of curvature $R = 5649$ mm; grating dimension $W \times L \times T = 50 \times 30 \times 10$ mm; parameters for space variation $b_2 = -8.9$; $b_3 = 86.3$; $b_4 = -1349$. (b) The calculated focal curve for the wavelength range of 22 – 124 nm. The result at 58.48 nm (21.2 eV) is marked by the diamond. The vertical dashed line marks the position of the spectral plane. (c) The calculated incident and outgoing light at 58.48 nm diffracted on the entire horizontal surface of the grating.
